# Quality Indicators in Pediatric Palliative Care: Considerations for Latin America

**DOI:** 10.3390/children8030250

**Published:** 2021-03-23

**Authors:** Gregorio Zuniga-Villanueva, Jorge Alberto Ramos-Guerrero, Monica Osio-Saldaña, Jessica A. Casas, Joan Marston, Regina Okhuysen-Cawley

**Affiliations:** 1 Department of Pediatrics, Tecnologico de Monterrey, Monterrey 64849, Mexico; 2 Division of Pediatric Palliative Medicine, McMaster University, Hamilton, ON L8N 3Z5, Canada; 3 Department of Palliative and Pain Medicine, Universidad de Guadalajara, Zapopan 45170, Mexico; paliativospediatricosmx@gmail.com; 4 Department of Global Studies, Universidad Nacional Autonoma de Mexico, Mexico City 04510, Mexico; osiomonicas@hotmail.com; 5 Department of Pediatric Palliative Care, Texas Children’s Hospital, Houston, TX 77030, USA; jxcasas@texaschildrens.org (J.A.C.); rxokhuys@texaschildrens.org (R.O.-C.); 6 Global Ambassador, International Children’s Palliative Care Network, Assagay 3624, South Africa; joanmarymarston@gmail.com

**Keywords:** quality indicators, pediatric palliative care, Latin America

## Abstract

Pediatric palliative care is a growing field in which the currently available resources are still insufficient to meet the palliative care needs of children worldwide. Specifically, in Latin America, pediatric palliative care services have emerged unevenly and are still considered underdeveloped when compared to other regions of the world. A crucial step in developing pediatric palliative care (PPC) programs is delineating quality indicators; however, no consensus has been reached on the outcomes or how to measure the impact of PPC. Additionally, Latin America has unique sociocultural characteristics that impact the perception, acceptance, enrollment and implementation of palliative care services. To date, no defined set of quality indicators has been proposed for the region. This article explores the limitations of current available quality indicators and describes the Latin American context and how it affects PPC development. This information can help guide the creation of standards of care and quality indicators that meet local PPC needs while considering the sociocultural landscape of Latin America and its population.

## 1. Introduction

Pediatric palliative care (PPC) is an interdisciplinary approach to care that focuses on maximizing quality of life (QoL) for children and their families who face a life-limiting or life-threatening illness. It starts at diagnosis and continues throughout the child’s life, during death and into bereavement. It embraces physical, emotional, social and spiritual elements while providing management of distressing symptoms, guidance through advanced care planning and goals of care, the provision of short breaks (respite), and grief support [1].

Worldwide, it is estimated that 4 million children need palliative care [2]. While the majority could benefit from generalized palliative care, the rest would still require specialized PPC [3]. Regarding the burden of serious health-related suffering (SHS), 5.3 million children experience SHS globally. SHS is defined as suffering associated with illness or injury of any kind that cannot be relieved without the intervention of a health care professional and when it compromises physical, social or emotional functioning. Since palliative care focuses on relieving SHS associated with serious health problems or the end of life, SHS can be used as a proxy for palliative care need [2,4].

This need has yet to be met, as accessibility to PPC continues to be disparate across the world, and PPC services are considered underdeveloped across Asia, Africa and Latin America (LA) compared to North America and Europe [2]. The growth of PPC services has typically been based on local resources and needs, usually due to highly motivated individuals, leading to different conformations in team structures and a diverse availability of services that they can provide [5]. The same is true for LA, where palliative care services have developed unevenly and without a defined pattern [6]. The presence of PPC in LA has grown in recent years; nonetheless, access to PPC in LA remains an unaddressed global health issue as efforts to improve palliative care development typically focus on adult services [7].

A crucial step in developing PPC programs is delineating quality indicators for PPC [8]. Various standards of care and guidelines have been developed to assess PPC impact and guide pragmatic development [9,10,11,12]. However, no consensus has been reached on the outcomes, quality indicators or how to measure the impact of PPC [13,14,15]. Furthermore, cultural preferences and structural differences in care provision influence PPC outcomes [16]. Culture, social norms and religious affiliations determine the perception, acceptance, enrollment and implementation of palliative care services [17].

In light of the immature stage of development of PPC in LA, the lack of consensus in PPC quality indicators and considering the impact that local culture has on the provision of palliative care, the objective of this paper is to review and describe specific characteristics of the LA context that uniquely influence PPC development. This article reflects on the factors that need to be considered when defining standards of care and quality indicators for PPC in LA.

## 2. Contextual Factors That Influence PPC Development in LA

To understand the nuances of the palliative care landscape in LA, specific historical, structural, cultural and legal components of LA will be addressed, along with a description of local factors that have influenced palliative care as a field and how these singularities have impacted the development and provision of PPC in the region.

### 2.1. Palliative Care Philosophy

Philosophically, there are core concepts unique to the palliative care ethos in LA and linguistic palliative care terms that do not have a seamless translation from English to Spanish and vice versa. The concept of therapeutic obstinacy, defined as the initiation or continuation of medical interventions and treatments that generate unnecessary suffering for the patient, with the goal of prolonging the patient’s life at all costs even when there is no reasonable hope for a cure, is considered to be the antithesis of palliative care philosophy [18]. This concept is widely mentioned in the Spanish palliative care literature, while it is seldom mentioned in the English literature.

Another concept that does not translate interchangeably is withholding and withdrawing life-sustaining treatments, as there is no specific translation for the words withdrawing and withholding that carry the same meaning across the languages. In turn, the Spanish literature uses the term adecuación del esfuerzo terapeútico, which also does not have a suitable translation in English. A literal translation would be adequacy of therapeutic effort, which can be interpreted as the patient’s right to receive adequate treatments in quantity and quality based on the patient’s clinical scenario [19]. This means that treatments and interventions should be modified and tailored based on the patient’s situation by not starting (withholding) or stopping them (withdrawal). The patient then receives an adequate amount of treatment: no more, and no less, just the right amount, proportional to the patient’s circumstances.

Advance care planning is another key term in palliative care that has conflicting translations in Spanish, as it is sometimes translated as advance decision planning [20] or advance directives [21], limiting the cohesive understanding of the concept. Additionally, the concept of goals of care is practically nonexistent in Spanish as there is not an equivalent translation or interchangeable concept that could be used in its place. These differences in core concepts in palliative care shape how palliative care is interpreted, taught and applied, possibly influencing how healthcare professionals and families alike believe in medical paternalism as beneficence and as the correct ethical framework in LA [22].

### 2.2. Healthcare Structure

Differences in the organization and funding of healthcare systems across LA countries have resulted in an unbalanced development of palliative care, making it challenging to create a unifying scheme that applies across borders [6]. Differences in health care systems stem from the ongoing relationship between public and private health services that coexist in many LA countries [23]. This creates inequitable access to healthcare, as some countries only offer private palliative care services usually paid out-of-pocket by their clients [6].

Service provision is limited to the almost absent presence of pediatric-specific hospices in LA and the scarce nursing homes or institutions that offer respite care [6]. This limits the capacity for families of medically complex children with palliative care needs to take respite breaks [24]. Other services that face constraints are home-care programs with knowledge on pediatric-specific needs [6].

### 2.3. Palliative Care Providers

Palliative care team structures are influenced by the healthcare professionals that comprise them. In some LA countries, palliative care services emerged as specialized hospital services that stemmed from pain-management clinics run by anesthesiologists [25]. This contrasts with the historical development of the hospice movement and its community approach [26] that evolved to lift Family Medicine as the core of palliative care programs [27]. Numerous palliative care departments include the word “pain” at the forefront of their names (i.e., Department of Pain and Palliative Medicine) [28], reflecting the origins of palliative care from pain medicine. In other LA countries, palliative care services are strictly focused on oncologic patients or are solely provided in cancer centers [29]. Moreover, many palliative care services, educational programs and palliative care specialty certifications are housed under anesthesiology, cancer or chronic illnesses that render their characterization difficult [6].

Some healthcare professionals that provide palliative care are either absent or exclusively present in LA. In other parts of the world, various palliative care programs have been spearheaded, developed, implemented and evaluated by advanced nurse practitioners [30]. In LA, there is a lack of regulation and an absence of a defined advanced practice nursing role [31]. This absence of nurse practitioners contrasts with the essential role that advanced practice nurses have in palliative care. Another healthcare professional who is absent from PPC teams in LA but has a growing presence in other parts of the world like North America is the child life specialist (CLS) [32]. CLSs are a group of health professionals who provide developmentally driven interventions to support pediatric patients, their parents and their siblings in order to help them cope, learn and understand their emotional and clinical condition [33]. CLSs have been considered core members of PPC teams as they can help children adapt to their new reality through their grief and support them in their end-of-life [32]. CLSs have yet to be introduced to PPC services in LA. In contrast, regarding grief and bereavement support, the field of thanatology (the study of loss, death and grief) has emerged in LA as the preferred field for offering grief counselling and support during and after the dying process [34]. Thanatologists, also known as end-of-life or death doulas, have been gradually incorporated into LA’s palliative care teams. The thanatologist’s figure has acquired a strong position in the provision of palliative care in LA [6]. The presence of thanatologists in palliative care teams is almost exclusive to LA.

### 2.4. Laws and Regulations in Palliative Care

The majority of LA countries do not have specific palliative care laws [6,29], and when present, they do not account for the particular needs of PPC. Laws are only effective when healthcare providers know and embrace them. It has been noted that despite pediatricians knowing PPC concepts, they do not feel comfortable addressing palliative care needs when they do not feel legally protected to offer anything different than cure-directed treatment [35]. Specifically for perinatal PC, abortion is still illegal in most LA countries [36], shifting the proportion of parents that face the birth of a child with life-limiting conditions and increasing the need for perinatal palliative care. This is in contrast to other parts of the world where perinatal palliative care services are offered to help families make decisions regarding the termination of pregnancy or to create a birth plan for pregnancies affected by a fetus with a life-limiting diagnosis [37].

## 3. Challenges of Implementing PPC Quality Indicators

Quality of care is the degree to which health services for individuals and populations increase the likelihood of desired health outcomes and are consistent with current professional knowledge [38]. Quality indicators are defined as standards that measure the structure, processes or outcomes of care for a particular type of patient or clinical circumstance [39]. Quality indicators in PPC should be based on the effectiveness of PPC at different levels that include patients, families, health-care professionals and health systems [14]. Nonetheless, current PPC standards of care reported in the literature face particular challenges that render their replicability across settings difficult [13,15]. It is imperative to detail these challenges that affect the PPC implementation of quality indicators.

### 3.1. Lack of Consensus

There is currently no ideal outcome assessment measure available in PPC [40]. Moreover, while improving QoL is considered the essence of PPC, an accurate description of how improving QoL is operationalized has yet to be provided [13]. More than 80 indicators in PPC have been described in the literature, with a wide variation in the detailed definitions across studies [14]. To simplify literature findings, various systematic reviews have attempted to group quality indicators and outcomes into themes, with varying degrees of similarity between the themes proposed by the different systematized reviews [14,15,41,42,43]. The disharmony in PPC standards also gets carried into how distinctively they are presented between multiple PPC organizations or programs [11,12,44,45,46,47,48]. For further reference, a list from a selected literature review of PPC standards of care (Table S1) [11,12,44,45,46,47,48] and quality indicators (Table S2) [14,15,41,42,43] are included as supplements.

### 3.2. Heterogenicity in PPC Patients

PPC encompasses more than 300 conditions classified as life-limiting or life-threatening in children [49]. This diversity is challenged by the many changing, unpredictable, complex needs that children with life-limiting conditions and their families face. PPC interventions require flexibility and adaptability to the multiple variations in illness trajectories [49], the wide range of children’s diseases and ages [13], differences in parental needs [50] and the difficulty in identifying a terminal stage in chronic-complex conditions [16]. This hinders the applicability of instruments that measure quality of life, as no instrument can capture the fluid needs of the complete diaspora of children with life-limiting conditions [51]. Additionally, some have suggested that families experiencing a life-limiting diagnosis during the perinatal period have specific needs that require a different set of quality indicators [37].

### 3.3. Selective Focus of the PPC Literature

Despite the vast array of diagnoses that could benefit from PPC, cancer continues to be the primary disease reflected in the PPC literature [16]. This disproportionally contrasts with the palliative care need in children as cancer only accounts for 4.1% of all the children that require PPC [2]. Much is still unknown regarding the nononcologic pediatric population and how to meet their palliative care needs. Furthermore, most studies lack the voices of children (patients and siblings) [14,15] or obtain their data from bereaved parents [43,49,50]. This introduces recall bias and an indirect interpretation of the events that might not reflect the patient’s experience. Likewise, PPC publications primarily focus on the care provided exclusively at the end-of-life [14,15], resulting in limited data on the impact of PPC when involved earlier in the disease trajectory.

## 4. Pathway towards Developing Quality Indicators for PPC in LA

Given that culture shapes the experience of illness, suffering and end-of-life [52], the currently available standards and quality indicators may not be entirely translatable for the LA context as they reflect the European and North American (Canada and United States) landscape, resources and mindset where these PPC services are offered. The available standards from other systems often represent an idealized version of what PPC should offer, which might seem unrealistic or unachievable for LA’s PPC services. Instead of replicating quality indicators verbatim, they should be adapted to meet the local needs of systems, patients, parents and siblings [49,50]. Nonetheless, rather than reinventing the wheel, PPC in LA can draw inspiration and guidance from international organizations and published literature to develop their own quality indicators.

Through a literature review, we summarized and categorized available PPC standards of care and quality indicators to match the level of development needed to achieve them (see Table 1) [11,12,14,15,41,42,43,44,45,46,47,48]. This list could be used as a reference to help PPC services in LA view the landscape of how these quality indicators and standards are applied internationally.

Quality indicators in PPC should be tailored to the level of development of PPC services and the location where they are applied. For example, it is unrealistic and unfair to expect the performance of a PPC champion from a particular system to deliver care that matches the quality standards of a fully developed team from a different system. Hence, as services continue to grow, teams can progressively target additional standards until PPC services reach their full potential within their system. It is essential to acknowledge that some indicators might require structural changes before they can be achieved, like promoting patient and family-centered care rather than a paternalistic approach, recognizing PPC as a subspecialty, increasing public awareness of PPC and consciously including PPC in healthcare professionals’ curricula.

Creating standards of care that apply PPC quality indicators in LA is dependent on the mapping of the level of development of PPC services in the region. While this task has been continuously performed by the International Children’s Palliative Care Network [53] and the Latin American Association of Palliative Care [6], there is still the need for a central repository to track the progress of PPC in order to plan an ongoing development [2].

Pragmatically, developing appropriate and feasible quality indicators that match the local sociocultural scenario will help PPC services meet the standards of care based on the available resources. Standards should reflect PPC services’ growth and developmental stage as palliative care interventions are broad and require a multidisciplinary approach [15]. The development of PPC programs in LA requires the co-design of services with the help of children and families with palliative care needs to reflect the local context, an essential component in convincing institutions of the necessity of PPC [9,41].

## 5. Conclusions

The development of PPC services in LA has yet to mature. As teams thrive and networks are formed, organized advocacy can help achieve the level of PPC that children in LA deserve. Future challenges that need to be tackled include the inclusion of governments in developing PPC services based on their local needs and resources. This paper is a call for action for interested parties to join forces to define standards of care and quality indicators that reflect the culture and population of LA.

## Figures and Tables

**Table 1:** Summary and categorization of standards of care and quality indicators based on the level of development of PPC.

Available Resources	Achievable Standards of Care	Description of Quality Indicators
Perinatal and PPC ^1^ champion or specialist	Child’s quality of life	Offers specialized pain and symptom management to achieve comfort and minimize total pain and suffering.
		Offers age-appropriate care during all disease stages.
		Uses patient-reported outcomes to guide care (when possible).
		Offers services to children with life-limiting and life-threatening conditions, which includes chronic-complex conditions, cancer and perinatal diagnosis.
	Family Support	Offers family-centered care by supporting the family unit.
		Helps families identify their goals.
		Encourages parents to be part of the care of the child.
	Communication	Offers clear and honest information to the child and family.
		Ensures communication with other healthcare providers who follow the patient.
	Advanced care planning	Promotes shared decision-making.
		Involves patients and families in developing treatment goals and plans.
		Helps families in difficult decision-making, including end-of-life care plans.
	End-of-life care	Provides care for the imminently dying patient.
		Assures that the intensity of treatment is in accordance with the goals of care and the child’s best interest.
		Helps families prepare for the death of the child.
	Referrals	Provides information about emotional and psychosocial support and how to access it.
		Provides information on bereavement support and how to access it.
	Education	Offers education to the family.
		Offers education to other healthcare professionals.
Specialized PPC team (it may include a combination of physicians, nursing, social worker, mental health specialist, child life specialist, spiritual support, grief counselling and other allied health professionals)	Holistic support	Offers multidisciplinary assessment and support of patient and family needs.
		Offers support to siblings and extended family.
		Offers psychological support to patient and family.
		Offers social support for patient and family.
		Offers spiritual support for patient and family.
		Offers expressive therapies including art, music, play and massage.
	Coordination of care	Designates a keyworker to the patient and family who leads and coordinates the care.
		Offers flexible delivery of care across settings to ensure continuity of care.
	Grief and bereavement support	Offers grief support before, during and after death.
		Offers continuing bereavement support for families during their grief process.
		Offers active grief support of siblings and extended family.
		Helps families make funeral arrangements.
	Availability	Offers access to PPC 24 h a day, seven days a week, 365 days a year.
	Healthcare professionals support	Promotes continuous training and self-care for all team members.
		Supports other healthcare professionals.
Community healthcare providers with PPC knowledge	Community provision of PPC	Provides palliative care and follow-up at home.
		Provides end-of-life care at home for families who desire it.
		Offers a seamless transition between the hospital and home.
Hospice	Respite	Offers respite for care providers to obtain brakes.
	End-of-life care	Offers end-of-life care outside of the hospital and in the community other than at home.
National Council, Association or College	Advocacy	Active lobbying for PPC development.
		Advocates for the compliance of laws and regulations.
	Research and publications	Development of national guidelines, standards or norms.
		Encourages local PPC providers to engage in research and academic activities.
	Education	Endorses perinatal and PPC training programs.
	Regulation	Assesses performance to ensure the quality of care provision.
		Regulates the professional activities of PPC providers.
	Collaboration	Creates a network that fosters collaboration between PPC providers.
		Creates partnerships between PPC and other specialties.
		Engages with international palliative care associations.
Health system	National law	Existence of law that protects PPC practice and recognizes specific pediatric needs in palliative care.
	Equity	Inclusive and equitable access to PPC across settings and institutions.
		Existence of specific pediatric formulations available in the national essential medicine list.
	Funding	Funding of palliative care services.
		Assessment of healthcare utilization and costs.
		Offer financial support to families with PPC needs.

^1^ PPC: pediatric palliative care.

## Data Availability

No new data were created or analyzed in this study. Data sharing is not applicable to this article.

## Supplementary Materials

The following are available online at https://www.mdpi.com/2227-9067/8/3/250/s1, Table S1: Selected list of standards of care in pediatric palliative care; and Table S2: Selected list of quality indicator’s themes in pediatric palliative care.

## References

[1] Chambers L. (2018). A Guide to Children’s Palliative Care.

[2] Connor S.R. (2020). Global Atlas of Palliative Care.

[3] Connor S.R., Downing J., Marston J. (2017). Estimating the Global Need for Palliative Care for Children: A Cross-Sectional Analysis. J. Pain Symptom Manag..

[4] Knaul F.M., Farmer P.E., Krakauer E.L., De Lima L., Bhadelia A., Jiang Kwete X., Arreola-Ornelas H., Gómez-Dantés O., Rodriguez N.M., Alleyne G.A.O. (2018). Alleviating the Access Abyss in Palliative Care and Pain Relief—an Imperative of Universal Health Coverage: The Lancet Commission Report. Lancet.

[5] Knapp C., Woodworth L., Wright M., Downing J., Drake R., Fowler-Kerry S., Hain R., Marston J. (2011). Pediatric Palliative Care Provision around the World: A Systematic Review. Pediatr. Blood Cancer.

[6] Pastrana L., De Lima L., Wenk R., Eisenchlas J., Monti C., Rocafort J., Centeno C. (2012). Atlas de Cuidados Paliativos de Latinoamérica ALCP.

[7] Garcia-Quintero X., Parra-Lara L.G., Claros-Hulbert A., Cuervo-Suarez M.I., Gomez-Garcia W., Desbrandes F., Arias-Casais N. (2020). Advancing Pediatric Palliative Care in a Low-Middle Income Country: An Implementation Study, a Challenging but Not Impossible Task. BMC Palliat. Care.

[8] Feudtner C. (2004). Perspectives on Quality at the End of Life. Arch. Pediatr. Adolesc. Med..

[9] Charlebois J., Cyr C. (2015). Quality Indicators for Paediatric Palliative Care. Paediatr. Child Health.

[10] National Institute for Health Care Excellence (NICE) (2016). NICE Guideline NG61: End of Life Care for Infants, Children and Young People with Life-Limiting Conditions: Planning and Management.

[11] National Hospice and Palliative Care Organization (NHPCO) (2019). Standards of Practice for Pediatric Palliative Care.

[12] European Association for Palliative Care TSG (last) (2007). IMPaCCT: Standards for Paediatric Palliative Care in Europe. Eur. J. Palliat. Care.

[13] Friedel M., Aujoulat I., Dubois A.-C., Degryse J.-M. (2019). Instruments to Measure Outcomes in Pediatric Palliative Care: A Systematic Review. Pediatrics.

[14] Widger K., Medeiros C., Trenholm M., Zuniga-Villanueva G., Streuli J.C. (2019). Indicators Used to Assess the Impact of Specialized Pediatric Palliative Care: A Scoping Review. J. Palliat. Med..

[15] Marcus K.L., Santos G., Ciapponi A., Comandé D., Bilodeau M., Wolfe J., Dussel V. (2020). Impact of Specialized Pediatric Palliative Care: A Systematic Review. J. Pain Symptom Manag..

[16] Wolff S.L., Christiansen C.F., Nielsen M.K., Johnsen S.P., Schroeder H., Neergaard M.A. (2020). Predictors for Place of Death among Children:A Systematic Review and Meta-Analyses of Recent Literature. Eur. J. Pediatr..

[17] Dosani N., Bhargava R., Arya A., Pang C., Tut P., Sharma A., Chasen M. (2020). Perceptions of Palliative Care in a South Asian Community: Findings from an Observational Study. BMC Palliat. Care.

[18] Zurriaráin R.G. (2020). Between Euthanasia and Therapeutic Obstinacy: Palliative Care. Hosp. Palliat. Med. Int. J..

[19] Pérez Pérez F.M. (2016). Adecuación del esfuerzo terapéutico, una estrategia al final de la vida. SEMERGEN Med. Fam..

[20] Diestre Ortín G., González Sequero V., Collell Domènech N., Pérez López F., Hernando Robles P. (2013). [Advance care planning and severe chronic diseases]. Rev. Espanola Geriatr. Gerontol..

[21] Martínez Gimeno M.L., Cámara Escribano C., Honrubia Fernández T., Olmo García M.C., Tovar Benito D.H., Bilbao-Goyoaga Arenas T., Rodríguez Almagro P., en nombre del grupo PLAMOS (2018). Knowledge and attitudes of health care professionals in advance healthcare directives. J. Healthc. Qual. Res..

[22] Machado K.K., Hoff P.M. (2012). Autonomy versus Paternalism in Latin America. Oncology (Williston Park NY).

[23] Bustamante A.V., Méndez C.A. (2014). Health Care Privatization in Latin America: Comparing Divergent Privatization Approaches in Chile, Colombia, and Mexico. J. Health Polit. Policy Law.

[24] Smith C.H., Graham C.A., Herbert A.R. (2017). Respite Needs of Families Receiving Palliative Care. J. Paediatr. Child Health.

[25] Covarrubias-Gómez A., Otero-Lamas M., Templos-Esteban L.A., Soto-Pérez-de-Celis E. (2019). Antecedentes de la medicina paliativa en México: Educación continua en cuidados paliativos. Rev. Mex. Anestesiol..

[26] Saunders C. (2001). The Evolution of Palliative Care. J. R. Soc. Med..

[27] Ramanayake R.P.J.C., Dilanka G.V.A., Premasiri L.W.S.S. (2016). Palliative Care; Role of Family Physicians. J. Fam. Med. Prim. Care.

[28] Lohman D., Herrera García A., Amon J., Wilkinson D., Kippenberg J., Reidy A., Olugboji B. (2014). Care When There Is No Cure: Ensuring the Right to Palliative Care in Mexico.

[29] Soto-Perez-de-Celis E., Chavarri-Guerra Y., Pastrana T., Ruiz-Mendoza R., Bukowski A., Goss P.E. (2017). End-of-Life Care in Latin America. J. Glob. Oncol..

[30] George T. (2016). Role of the Advanced Practice Nurse in Palliative Care. Int. J. Palliat. Nurs..

[31] Zug K.E., Cassiani S.H.D.B., Pulcini J., Garcia A.B., Aguirre-Boza F., Park J. (2016). Advanced Practice Nursing in Latin America and the Caribbean: Regulation, Education and Practice. Rev. Lat. Am. Enfermagem.

[32] Basak R.B., Momaya R., Guo J., Rathi P. (2019). Role of Child Life Specialists in Pediatric Palliative Care. J. Pain Symptom Manag..

[33] Sutter C., Reid T. (2012). How Do We Talk to the Children? Child Life Consultation to Support the Children of Seriously Ill Adult Inpatients. J. Palliat. Med..

[34] Färber S.S. (2013). Tanatologia clínica e cuidados paliativos: Facilitadores do luto oncológico pediátrico. Cad. Saúde Coletiva.

[35] Zuniga-Villanueva G., Ramirez-GarciaLuna J.L., Weingarten K. (2019). Factors Associated With Knowledge and Comfort Providing Palliative Care: A Survey of Pediatricians in Mexico. J. Palliat. Care.

[36] Zúñiga-Fajuri A. (2014). Human Rights and the Right to Abortion in Latin America. Cienc. Saude Coletiva.

[37] Wool C. (2015). Instrument Development: Parental Satisfaction and Quality Indicators of Perinatal Palliative Care. J. Hosp. Palliat. Nurs..

[38] Organisation for Economic Co-operation and Development, World Health Organization, World Bank Group (2018). Delivering Quality Health Services: A Global Imperative for Universal Health Coverage.

[39] Donabedian A. (1988). The Quality of Care. How Can It Be Assessed?. JAMA.

[40] Coombes L.H., Wiseman T., Lucas G., Sangha A., Murtagh F.E. (2016). Health-Related Quality-of-Life Outcome Measures in Paediatric Palliative Care: A Systematic Review of Psychometric Properties and Feasibility of Use. Palliat. Med..

[41] Mitchell S., Morris A., Bennett K., Sajid L., Dale J. (2017). Specialist Paediatric Palliative Care Services: What Are the Benefits?. Arch. Dis. Child..

[42] Zuniga-Villanueva G., Widger K., Medeiros C., Trenholm M., Streuli J.C. (2020). Specialized Pediatric Palliative Care in Neonates with Life-Limiting Illness: A Systematic Review. Am. J. Perinatol..

[43] Widger K.A., Wilkins K. (2004). What Are the Key Components of Quality Perinatal and Pediatric End-of-Life Care? A Literature Review. J. Palliat. Care.

[44] Together for Short Lives (2015). Standards Framework for Children’s Palliative Care.

[45] National Instituce for Health and Care Excellence (2017). NICE Quality Standard QS160: End of Life Care for Infants, Children and Young People.

[46] Gans D., Kominski G.F., Roby D.H., Diamant A.L., Chen X., Lin W., Hohe N. (2012). Better Outcomes, Lower Costs: Palliative Care Program Reduces Stress, Costs of Care for Children with Life-Threatening Conditions.

[47] Bergstraesser E., Zimmermann K., Eskola K., Luck P., Ramelet A.-S., Cignacco E. (2015). Paediatric End-of-Life Care Needs in Switzerland: Current Practices, and Perspectives from Parents and Professionals. A Study Protocol. J. Adv. Nurs..

[48] Ferrell B.R., Twaddle M.L., Melnick A., Meier D.E. (2018). National Consensus Project Clinical Practice Guidelines for Quality Palliative Care Guidelines, 4th Edition. J. Palliat. Med..

[49] Constantinou G., Garcia R., Cook E., Randhawa G. (2019). Children’s Unmet Palliative Care Needs: A Scoping Review of Parents’ Perspectives. BMJ Support. Palliat. Care.

[50] Leemann T., Bergstraesser E., Cignacco E., Zimmermann K. (2020). Differing Needs of Mothers and Fathers during Their Child’s End-of-Life Care: Secondary Analysis of the “Paediatric End-of-Life Care Needs” (PELICAN) Study. BMC Palliat. Care.

[51] Huang I.-C., Shenkman E.A., Madden V.L., Vadaparampil S., Quinn G., Knapp C.A. (2010). Measuring Quality of Life in Pediatric Palliative Care: Challenges and Potential Solutions. Palliat. Med..

[52] Wiener L., McConnell D.G., Latella L., Ludi E. (2013). Cultural and Religious Considerations in Pediatric Palliative Care. Palliat. Support. Care.

[53] Marston J., Boucher S., Downing J. (2018). International Children’s Palliative Care Network: A Global Action Network for Children With Life-Limiting Conditions. J. Pain Symptom Manag..

