# Supplementary material for: Quality Indicators in Pediatric Palliative Care: Considerations for Latin America
Source: Children (Basel). 2021 Mar 23;8(3):250. doi: 10.3390/children8030250 (PMC8004984; doi:10.3390/children8030250)
Supplement: Supplementary file 1 [file children-08-00250-s001.pdf]

Supplementary Materials

Table S1. Selected list of standards of care in pediatric palliative care.

**Together for Short Lives**	**National Institute for Health and Care Excellence**	**Partners for Children Program**	**Paediatric End-of-Life Care Needs (PELICAN) study**	**National Consensus Project for Quality Palliative Care**	**National Hospice and Palliative Care Organization**	**European association of palliative care**
Sharing prognosis and significant news	Involve patients and families in developing an advanced care plan	Care coordination across settings	Support for the family unit	Structure and processes of care	Clinical excellence and safety: pursue comfort and minimize total pain and suffering	Provision of care across settings during all disease stages
Seamless transition between hospital and community care	Have a medical specialist who leads and coordinates their care	Expressive therapies including art, music, play and massage	Communication with the child and the family about treatment goals and plans	Physical aspects of care	Compliance with laws and regulation	Child and family as the unit of care
Multi-disciplinary assessment of needs	Receive information about emotional and psychosocial support and how to access it	Family education	Ethics and shared decision-making	Psychological and psychiatric aspects of care	Ethical behavior and consumer rights: clinical decisions guided by the best interest of the child	Care team with a holistic approach
A child and family care plan	Receive care by a multidisciplinary team that includes specialized PPC[1]	Respite	Symptom management	Social aspects of care	Inclusion and access: equitable access to care	Care coordinator / keyworker designated to patient and family
End of life plan	Receive support for grief and loss before and after death	Family and bereavement counseling	Continuity of care	Spiritual, religious, and existential	Organizational excellence: flexible delivery of	Symptom management available 24/7

				aspects of care	care across settings	
Continuing bereavement support	Have 24-hour access to PPC	Pain and symptom management available 24/7	Grief and bereavement support	Cultural aspects of care	Patient and family-centered care	Respite care
				Care of the imminently dying patient	Performance measurement: continuous quality assessment of care	Bereavement support before and after death
				Ethical and legal aspects of care	Stewardship and accountability: development of policies	Age-appropriate care
					Workforce excellence: training and self-care for all team members	Education and training for professional and volunteers
						Funding for palliative care services

[1] PPC: pediatric palliative care.

Table S2. Selected list of quality indicator's themes in pediatric palliative care.

**Mitchell et al., 2017**	**Marcus et al., 2020**	**Zuniga-Villanueva et al., 2020**	**Widger et al., 2004**	**Widger et al., 2019**
Quality of Life	Patient symptoms and quality of life	Intensity of treatment	Information transmission	Health care utilization
Symptom control	Caregiver outcomes	Psychological, social, and spiritual support	Preparation for death and care at death	Location of death
Place of care	End-of-life discussions	Communication	Bereavement care	Cost

Family support	Patterns of end-of-life care	Location of Care	Interpersonal aspects and competence of health professionals	Child's quality of life
	Health care utilization	Symptom management	Clarity and honesty of communicati on	Child's symptom assessment and management
	Utilization of supportive services	Bereavement care	Parent Role	Procedures
		Predicted versus real neonatal outcomes	Support for the family	Family health
		Parental coping, stress and satisfaction	Pain and symptom management	Family support
			Decision making	Communicat ion with healthcare providers
			Psychosocial and spiritual needs of child	End-of-life care discussions and planning
			Availability and accessibility	Quality of care provision
			Coordination and integration	Bereavement follow up
			Respite care	Effect on health care providers
			Financial arrangement s	Others
